# Parental knowledge and awareness of complications of recurrent adenotonsillitis and its surgical management in children in the Jazan region, Saudi Arabia: a cross-sectional study

**DOI:** 10.3389/fped.2026.1761634

**Published:** 2026-04-15

**Authors:** Abdulelah A. Otaif, Ramzi M. Dighriri, Abdulrahman A. Otaif, Riyadh A. Jahlan, Osama A. Alotayf, Ali Y. Shaikh, Saud N. Alwadani, Raghad M. Alnami, Asma M. Soweedi

**Affiliations:** 1 Faculty of Medicine, Jazan University, Jazan, Saudi Arabia; 2 Department of Otolaryngology-Head & Neck Surgery, Armed Forces Hospital, Jazan, Saudi Arabia

**Keywords:** adenoid hypertrophy, adenotonsillectomy, jazan region, parental knowledge, pediatric ENT, recurrent adenotonsillitis, Saudi arabia, tonsillectomy complications

## Abstract

**Background:**

Recurrent adenotonsillitis is a frequent pediatric condition with complications affecting sleep, growth, hearing, and behavior. Parental awareness is essential for early identification and timely intervention.

**Aim:**

This study aimed to assess parental knowledge and awareness of recurrent adenotonsillitis complications and its surgical management among parents in the Jazan region, Saudi Arabia.

**Methods:**

A cross-sectional survey was conducted among parents residing in the Jazan region who had at least one child aged 1–14 years. A validated self-administered questionnaire assessed sociodemographic characteristics and knowledge of adenotonsillar disease and surgery. Descriptive and inferential statistical analyses were performed using SPSS version 26, with significance set at *p* < 0.05.

**Results:**

Out of 448 respondents, 311 met inclusion criteria. Most were female (59%), Saudi (97%), and had university-level education (72%). About 57% of parents showed good knowledge based on scoring above the mean. Awareness was higher regarding symptoms like snoring (66%), sleep apnea (67%), and voice changes (75%). However, fewer parents recognized complications such as pulmonary hypertension (28%), GERD (32%), or dental issues (31%). Nearly half (49%) acknowledged weight loss as a possible consequence of chronic infections, and 58% agreed neck swelling could be a sign. Regarding surgery, 44% believed immunity might weaken post-adenotonsillectomy, and 47% identified bleeding as the most common complication. Only 40% were aware of adenoid regrowth. Age over 40 (*p* < 0.001) and positive family history (*p* = 0.001) were significantly associated with higher knowledge.

**Conclusion:**

While parental awareness of some aspects of adenotonsillar complications is adequate, notable gaps remain, particularly concerning long-term and systemic effects. Targeted health education campaigns are essential to bridge these gaps and support better pediatric health outcomes.

## Introduction

The lymphatic system, which includes the tonsils and adenoids, effectively eliminates infection sources that enter the body through the mouth and nose and preserves bodily fluid equilibrium (1). Moreover, the tonsils and adenoids are vital components of the mucosal immune defense system against antigens entering through the aerodigestive tract and have basic roles in the humoral and cellular immune systems (2).

Tonsillitis is a prevalent illness in both pediatric and otolaryngology patients. Its treatment frequently entails administering empirical antibiotics without waiting for culture results. Antibiotic resistance, caused by *β*-lactamase generation and other reasons, can lead to recurrent or chronic tonsillitis (3).

Long-term adenotonsillar enlargement in children can result in obstructive sleep apnea, which can cause a number of complications, including poor academic performance, irritable behavior, and fragmented sleep. It can also cause pulmonary hypertension and congestive heart failure in the extremities, which can cause recurrent or chronic tonsillitis (4). Adenotonsillar hypertrophy-induced mouth breathing resulted in misaligned teeth and malocclusion, among other dentofacial changes (5). Adenoid hypertrophy can cause rhinosinusitis by manually blocking secretions or acting as a harbor for harmful microorganisms (6). Adenoid hypertrophy is associated with otitis media with effusion, which results in silent, progressive hearing loss in children that impacts behavior and speech (7).

Recurrent tonsillitis is defined as having more than seven episodes of tonsillitis in one year, more than five episodes each year for more than two years, or more than three episodes per year for more than three years (8).

One of the most common surgical operations on children is an adenotonsillectomy, which is frequently done as a day case. Tonsillectomies still make about 20% of all otolaryngologists' procedures, even if their number has decreased in recent years (9). Recurrent infections, tonsillar abscess, adenoid and obstructive sleep apnea, and tonsil enlargement are the most frequent reasons for adenotonsillectomy (10, 11). Recurrent ear infections, hearing loss, chronic sinusitis, terrible mouth breathing, or a tumor in the throat or nasal canal are additional reasons for an adenotonsillectomy (12, 13).

Recent studies in Saudi Arabia have highlighted a significant lack of parental knowledge regarding adenotonsillar diseases and their complications in children. In Al Madinah, 43.2% of parents had poor knowledge about recurrent adenotonsillitis complications and surgical treatment (14). Similarly, in the Aseer region, while 71.4% of parents correctly identified tonsil and adenoidal enlargement as a complication, there was still a need for additional education (15). A nationwide study found that only 6.2% of parents had good knowledge about adenoid hypertrophy and its orthodontic complications (16). These findings underscore the urgent need for targeted educational programs to improve parental awareness and understanding of adenotonsillar diseases, their complications, and management options in Saudi Arabia.

In the Jazan region of Saudi Arabia, there is a notable lack of studies investigating parental knowledge and awareness about recurrent adenotonsillitis and its surgical management. Since parents are both decision-makers and primary caregivers in pediatric care, understanding their perceptions and experiences—particularly regarding postoperative recovery—is vital for optimizing healthcare delivery. This study aims to assess parental awareness of the complications of recurrent adenotonsillitis and its surgical treatment. The findings will contribute to improving parental education, facilitating early diagnosis, and enhancing doctor-parent communication. Moreover, they will inform public health initiatives, medical training, and policy decisions to improve healthcare access and specialist availability in the Jazan region. Ultimately, this research will serve as a foundation for future interventions aimed at boosting parental awareness and improving pediatric healthcare outcomes.

## Methods

### Study design and setting

This study was a cross-sectional, questionnaire-based survey conducted in the Jazan region of Saudi Arabia from January to June 2025. The study aimed to evaluate the level of parental knowledge and awareness regarding the complications of recurrent adenotonsillitis and its surgical treatment.

### Study population

The study targeted parents residing in the Jazan region who were 18 years of age or older and had at least one child aged between 1 and 14 years during the study period. Only Arabic-speaking individuals who provided informed consent were included. Those who lived outside the region, were under 18 years old, did not have children in the specified age group, or were non-Arabic speakers were excluded from participation.

### Sampling and sample size

A convenience (non-probability) sampling technique was used to recruit participants for this study. The data were collected using an online self-administered questionnaire distributed via popular social media platforms, including WhatsApp, Telegram, and X (formerly Twitter). The questionnaire link was shared with initial participants, who were encouraged to forward it to others fitting the inclusion criteria. The required sample size was calculated using the Raosoft online sample size calculator (http://www.raosoft.com/samplesize.html), based on an estimated population size of 1,404,997 individuals in the Jazan region. With a 95% confidence level, a 5% margin of error, and an assumed response distribution of 50%, the minimum required sample size was 385 participants. To account for potential non-responses or ineligible entries, the target sample size was increased to 424. A total of 448 responses were collected. However, 114 participants were excluded for not having children aged between 1 and 14 years, and an additional 23 participants were excluded for not residing in the Jazan region. These criteria were specified *a priori* in the study's inclusion and exclusion criteria. After applying these exclusions, the final sample consisted of 311 eligible participants, which was used for the final analysis.

### Instrument for data collection

Data were collected using a self-administered, pre-validated questionnaire that was adapted with permission from a previous study (15). The questionnaire included items assessing participants’ sociodemographic information (age, gender, nationality, and education) and their knowledge of adenotonsillar function, potential complications of recurrent infections, and possible complications of adenotonsillectomy. The internal consistency of the questionnaire was evaluated using Cronbach's alpha. A pilot study was conducted on 30 participants to assess the clarity and usability of the instrument, and these individuals were excluded from the final analysis.

### Ethical considerations

This study was conducted in accordance with ethical principles governing research involving human participants. Ethical approval was obtained from the Institutional Review Board (IRB) of Jazan University (Approval No. REC-46/09/1463). All participants were informed about the purpose, methodology, and voluntary nature of the study, and informed consent was obtained prior to participation. Participants were assured of their right to withdraw from the study at any time without any consequences. All data were collected anonymously and handled with strict confidentiality. No personally identifiable information was recorded, and all responses were securely stored and used solely for research purposes.

### Statistical analysis

Data was gathered in Microsoft Excel, cleaned, and analyzed using SPSS version 26. The normality of continuous variables was assessed using histograms and Kolmogorov–Smirnov test. Descriptive statistics included frequencies and percentages for categorical variables and mean with standard deviation for continuous variables. For knowledge score, correct answers were coded one, incorrect and insured answers were coded zero, the total score was calculated. Participants who scored ≥ the mean value were considered to have good knowledge. Pearson's Chi-squared and Fisher's exact tests were performed to identify predictors of knowledge. Statistical significance was set at *P* < 0.05.

## Results

The study included 311 parents with the majority aged 30–40 years. Most participants were females (59%), Saudi (97%) and had university education (72%). Most participants reported positive family history of recurrent adenotonsillitis Table 1.

**Table 1:** Demographic characteristics of study participants.

Characteristic	*N* = 311^a^
Age	
18–29	68 (22%)
30–40	136 (44%)
More than 40	107 (34%)
Gender	
Female	183 (59%)
Male	128 (41%)
Nationality	
Non-Saudi	8 (2.6%)
Saudi	303 (97%)
Education	
Illiterate	2 (0.6%)
Primary/Secondary/High school education	84 (27%)
University education	225 (72%)
Family history of recurrent adenotonsillitis	
No	
Yes	188 (60%)

a n (%).

Regarding recurrent infections could lead to enlargement of the tonsils and adenoids, 70% agreed, 8.7% disagreed, and 21% reported uncertainty. Nearly half (49%) agreed that adenoids and tonsils are lymphoid tissues, 10% disagreed, and 41% were unsure, while 43% agreed that enlarged adenoids affect overall growth, 14% disagreed, and 43% indicated they did not know. Similarly, 41% agreed that enlarged adenoids could affect facial growth and contribute to jaw protrusion and dental misalignment.

Fifty nine percent agreed, 8.4% disagreed, and 33% were unsure if the middle ear infections improve following tonsillectomy and adenoidectomy. A majority (66%) agreed that enlarged tonsils and adenoids are associated with snoring, while 67% agreed that these structures could contribute to sleep apnea. Additionally, 75% agreed that enlarged tonsils and adenoids could cause voice changes; 5.8% disagreed, and 20% were uncertain.

In relation to halitosis, 73% agreed that it may result from chronic infections, however 31% agreed that infections could contribute to dental caries. Regarding weight loss as a potential consequence of recurrent infections, 49% agreed, 13% disagreed, and 39% reported uncertainty.

With respect to the association between chronic tonsillitis/adenoiditis and systemic complications such as pulmonary hypertension and heart failure, 28% agreed, 13% disagreed, and 60% were unsure. Nearly a third (32%) agreed that recurrent tonsillar and adenoid infections may be linked to gastroesophageal reflux disease (GERD). Additionally, 58% agreed that neck swelling could be a clinical presentation.

However, 41% disagreed with performing tonsillectomy and adenoidectomy during active infection, 24% agreed, and 34% were unsure. Regarding immune function following removal, 44% believed that immunity might weaken postoperatively, 16% disagreed, and 40% were uncertain. As for the possibility of obesity or weight gain as complications of removal, 32% agreed, 20% disagreed, and 48% were unsure.

**Figure 1:**
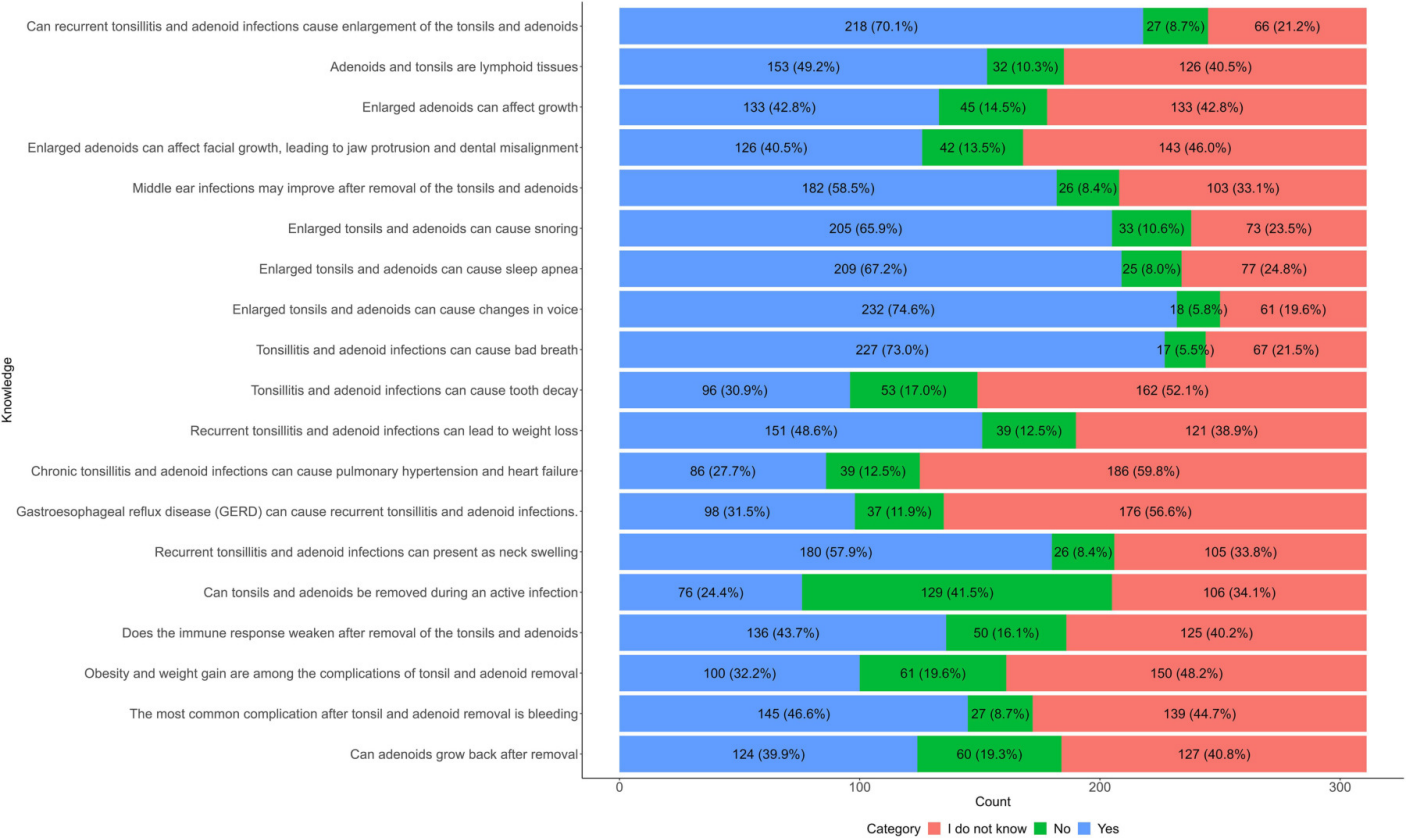
Knowledge of parents regarding recurrent adenotonsillitis.

Forty seven percent agreed that bleeding is the most common complication of tonsillectomy/adenoidectomy, 8.7% disagreed, and 45% were unsure. Forty percent agreed, 19% disagreed, and 41% were uncertain about adenoid regrowth after surgical removal. Figure 1.

**Figure 2:**
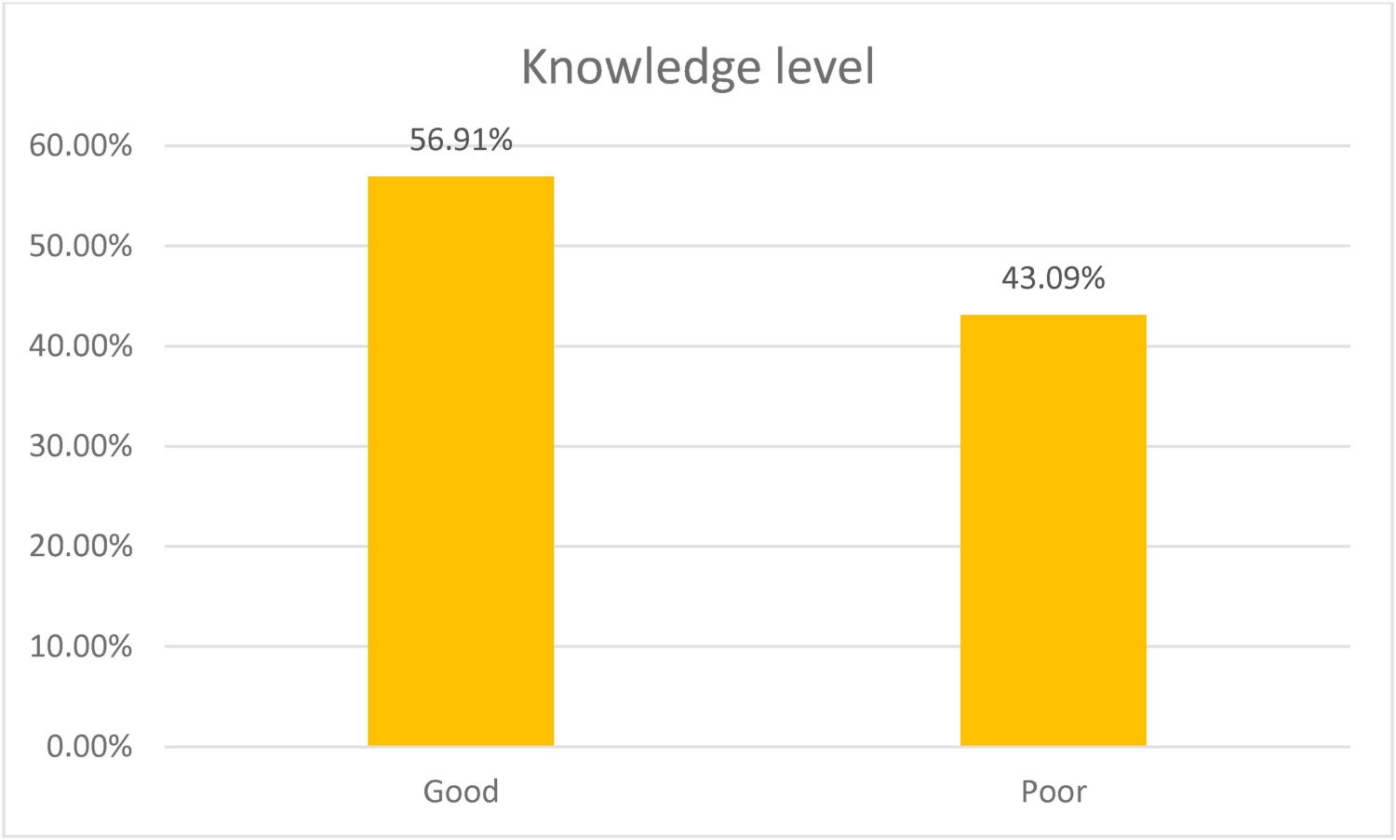
Knowledge level regarding recurrent adenotonsillitis.

Approximately 57% of parents had good knowledge about recurrent adenotonsillitis in their children Figure 2.

Only age (*p* < 0.001) and family history with recurrent adenotonsillitis (*p* = 0.001) were associated with knowledge regarding recurrent adenotonsillitis. Participants who aged more than forty years had a higher knowledge than younger ones. Similarly, those with family history with recurrent adenotonsillitis had higher knowledge than those with no family history. Table 2.

**Table 2:** Determinants of knowledge regarding recurrent adenotonsillitis among parents.

Characteristic	Poor, *N* = 134^a^	Good, *N* = 177^a^	*p*-value^b^
Age			**<0**.**001**
18–29	41 (31%)	27 (15%)	
30–40	61 (46%)	75 (42%)	
More than 40	32 (24%)	75 (42%)	
Gender			0.7
Female	77 (57%)	106 (60%)	
Male	57 (43%)	71 (40%)	
Nationality			0.3
Non-Saudi	5 (3.7%)	3 (1.7%)	
Saudi	129 (96%)	174 (98%)	
Education			0.4
Illiterate	1 (0.7%)	1 (0.6%)	
Primary/Secondary/High school education	41 (31%)	43 (24%)	
University education	92 (69%)	133 (75%)	
Family history of recurrent adenotonsillitis			**0**.**001**
No	67 (50%)	56 (32%)	
Yes	67 (50%)	121 (68%)	

a n (%).

b Pearson's Chi-squared test; Fisher's exact test.

## Discussion

Parental knowledge of recurrent adenotonsillitis and its surgical treatment in Jazan was investigated in our study. Although symptom knowledge was moderate, we discovered that there was a markedly lower level of awareness regarding major complications and surgical outcomes. A family history and advanced age were found to be powerful indicators of greater knowledge, indicating that demographic characteristics are crucial in determining parental comprehension.

Our findings are corroborated by a number of Saudi studies. Similar to our findings that 57% of parents showed good knowledge, Felemban et al. in Al-Madinah reported that 43% of parents lacked adequate knowledge regarding postoperative complications, with greater understanding being correlated with higher education and family history (14). Likewise, Alzahrani et al. discovered that only 6.2% of parents across the country were well-informed about orthodontic issues resulting from adenoid hypertrophy, indicating pervasive informational deficiencies (16). A study from the Aseer region found that the majority of parents correctly identified enlarged tonsils and adenoids as causes of snoring (89.4%) and sleep apnea (81.4%), which supports our findings of high parental awareness of these symptoms, with 66% and 67% recognizing snoring and sleep apnea, respectively (15).

The prevalence of symptoms like snoring and sleep apnea is probably high because these symptoms are obvious and disruptive, which makes it simpler for parents to spot them. Better general knowledge may also have resulted from participants' high educational attainment. Nonetheless, the lack of knowledge regarding complications like GERD and pulmonary hypertension raises the possibility that parents are not being adequately informed during clinical visits. A lack of counseling time or an emphasis on short-term issues rather than long-term results could be the cause of this. 44% of parents believe that adenotonsillectomy reduces immunity; this belief may be influenced by cultural misconceptions and false information, especially from social media. Furthermore, the correlation between greater knowledge and advanced age or family history highlights the importance of early and thorough education for all parents and suggests that experience is a major factor in forming awareness.

The validity of our findings is further reinforced by the strong support of prior regional and international studies. In our study, 57% of parents displayed good knowledge regarding recurrent adenotonsillitis and its related complications. This is consistent with the findings of a Madinah research, which found that parents with higher education levels or a family history of adenotonsillitis had greater knowledge than others (14). Similarly, a Tanzanian study discovered that, while 82.3% of parents recognized the overall impact of recurrent tonsillitis on their child's health, less than half were aware of specific consequences such as hearing loss, sleep apnea, or surgical dangers (17).

In our study, family history was substantially linked with higher knowledge (*p* = 0.001), which is consistent with the findings from both the Madinah and Aseer region studies, where personal or familial experience with adenotonsillitis increased parental awareness (14, 15). Furthermore, misunderstandings about immune function following adenotonsillectomy were common in our group, with 44% of parents assuming that immunity may deteriorate after surgery and 40% uncertain. This was also found in Abha and Qassim, where adults were concerned about their lowered immunity after surgery (18, 19).

In terms of surgical awareness, a considerable number of our participants were unclear of key facts, with 45% wondering if bleeding was the most common consequence and 34% unsure whether surgery could be conducted while the infection was active. These deficits are consistent with studies from Hofuf, where low perioperative education by healthcare professionals was connected to poor parental comprehension of surgical risks, notably bleeding (20). Interestingly, despite the fact that 72% of our participants had a university degree, education level was not significantly linked with knowledge (*p* = 0.4). This suggests that general education alone is insufficient to achieve good health literacy, a result confirmed by the Aseer research and international findings which emphasize the importance of disease-specific education (15, 21). Overall, these comparisons confirm that our findings are consistent with previous research and emphasize the importance of focused parental education efforts to address particular misunderstandings and enhance surgical decision-making.

Our study showed that although parental awareness was generally adequate, several misconceptions were still prevalent, which is different from numerous regional and international studies. For instance, fewer parents recognized facial growth abnormalities (41%) and dental malalignment (31%), despite the fact that 66% of our participants correctly linked snoring to adenotonsillar hypertrophy. This contrasts with the findings of Kimario et al. (17), who reported that even though 82.3% of Tanzanian parents recognized the general impact of adenotonsillitis, awareness of complications like hearing loss or sleep-related problems was much lower (17). Furthermore, only 28% of participants in our study were aware of the risk of pulmonary hypertension, whereas Qassim had a higher awareness of systemic sequelae such sleep apnea and heart-related problems, according to Alenezi et al. (19). These discrepancies can be due to different access points to pediatric ENT experts or regional health education initiatives.

In the perception of surgical timing and indications, our findings show 41% of parents disagreed with performing tonsillectomy and adenoidectomy during active infection, and 34% were uncertain. This reflects a high degree of confusion, which was less evident in Alkhars et al. (20), who found that parents in Hofuf were more likely to follow physician guidance regarding surgical timing (20). Additionally, although both studies showed uncertainty regarding post-tonsillectomy immunity, Al-Shaikh et al. (18) in Abha reported that parents were more confident in their understanding of surgical risks due to stronger counseling by surgeons (18). In contrast, our findings may indicate a communication gap between ENT providers and parents in the Jazan region, potentially due to time constraints or lack of accessible educational materials.

Additionally, despite the fact that our study found no significant correlation between parental knowledge and education level (*p* = 0.4), other previous studies found that education was a powerful predictor. For example, among university graduates in the study of Felemban et al. (14), education level was substantially associated with knowledge (*p* < 0.001) (14). Similarly, Musleh et al. (15) reported that higher educational attainment correlated with increased awareness of adenotonsillitis complications in the Aseer region (15). This discrepancy implies that, in some populations, academic achievement alone might not be a good predictor of health literacy, especially in the absence of disease-specific counseling. It also suggests that other factors, such cultural attitudes, healthcare professional communication, or personal or family experience, might have a greater influence on our sample's knowledge.

### Implications

The results of this study show that parents in the Jazan region have a moderate general awareness of recurrent adenotonsillitis and how to treat it. However, there are notable gaps in their knowledge of long-term and systemic complications, including dental malocclusion, GERD, and pulmonary hypertension. Parental ambiguity about surgical outcomes, postoperative dangers, and the possibility of adenoid regrowth was evident despite a reasonably high level of symptom identification, especially with relation to snoring, sleep apnea, and voice abnormalities.

It is clear that lived experience shapes comprehension because older parents and those with a family history of adenotonsillitis knew a lot more. This implies that younger or first-time parents might not be as prepared to spot warning signs or seek early help. Adenotonsillectomy-related misconceptions, such the idea that it impairs immunity, are another example of how cultural narratives and false information can have an impact on treatment choices.

Public health policy, health communication, and clinical practice are all directly impacted by these findings. It is obvious that communication between the doctor and the parent needs to be improved during consultations. This includes discussing potential consequences as well as the reasons for surgery when necessary, in addition to the current symptoms.

### Limitations

Nonetheless, certain limitations should be noted. The reliance on convenience sampling may affect the generalizability of the findings, and the self-reported nature of the data introduces potential biases. The cross-sectional design limits the ability to draw causal relationships, and the method used to classify knowledge may not accurately capture the depth of participants' understanding. Moreover, the absence of qualitative data and exploration of additional influencing factors restricts insight into the underlying reasons for prevalent misconceptions.

### Recommendations

Based on the findings of this study, several targeted strategies are recommended to improve parental awareness of recurrent adenotonsillitis and its management. Culturally appropriate health education campaigns should be implemented across the Jazan region, with particular emphasis on reaching parents under the age of 40, to address the full spectrum of adenotonsillar complications and the benefits and risks of surgical intervention. Primary care and pediatric clinics should be equipped with standardized educational materials such as brochures, videos, and infographics that can be used during consultations to dispel common misconceptions, including the belief that tonsillectomy weakens immunity. In addition, school-based health sessions and community workshops, conducted in collaboration with local healthcare authorities and ENT specialists, can serve as accessible platforms for parental education. Given the high digital engagement in the region, social media platforms like Instagram, X (formerly Twitter), and WhatsApp should be leveraged to disseminate accurate, evidence-based information in Arabic. Structured preoperative counseling should also be provided to parents of children undergoing adenotonsillectomy, covering realistic expectations for recovery, potential risks, and outcomes—including less common issues such as adenoid regrowth. Furthermore, national health promotion initiatives, particularly those aligned with Saudi Vision 2030, should incorporate ENT-related awareness to ensure broader public engagement. Lastly, future research should explore the primary sources of health information trusted by parents and evaluate the long-term effectiveness of various educational interventions in improving both knowledge retention and informed clinical decision-making. Future studies are recommended to employ multivariable logistic regression analysis to adjust for potential confounding variables such as age, gender, education level, and family history, in order to better identify independent predictors of knowledge.

## Conclusion

This study revealed a moderate level of parental knowledge regarding the complications of recurrent adenotonsillitis and its surgical management among residents of the Jazan region. While a majority of parents demonstrated awareness of common symptoms and consequences—such as snoring, sleep apnea, and voice changes—significant gaps remain in understanding less obvious yet serious complications, including potential impacts on systemic health and postoperative outcomes. Notably, older age and a positive family history were associated with higher knowledge levels. These findings underscore the need for targeted educational interventions to enhance parental awareness and promote timely medical consultation and treatment. By addressing these knowledge gaps, healthcare professionals can support better health outcomes for children affected by recurrent adenotonsillar conditions in the region.

## Data Availability

The original contributions presented in the study are included in the article/Supplementary Material, further inquiries can be directed to the corresponding author/s.
